# Recent Advances in Assessment and Treatment in Kienböck’s Disease

**DOI:** 10.3390/jcm11030664

**Published:** 2022-01-27

**Authors:** Karol Chojnowski, Mikołaj Opiełka, Miłosz Piotrowicz, Bartosz Kamil Sobocki, Justyna Napora, Filip Dąbrowski, Maciej Piotrowski, Tomasz Mazurek

**Affiliations:** Department of Orthopaedics and Traumatology, Faculty of Medicine, Medical University of Gdansk, 80-803 Gdansk, Poland; karole97@gumed.edu.pl (K.C.); mopielka@gumed.edu.pl (M.O.); piotrowicz.milosz@gumed.edu.pl (M.P.); b.sobocki@gumed.edu.pl (B.K.S.); justanapora@gmail.com (J.N.); maciejpiotr@gumed.edu.pl (M.P.); mazurek@gumed.edu.pl (T.M.)

**Keywords:** Kienböck, wrist, lunate bone, osteonecrosis

## Abstract

Kienböck’s disease is a rare disease described as progressive avascular osteonecrosis of the lunate. The typical manifestations include a unilateral reduction in wrist motion with accompanying pain and swelling. Besides recent advances in treatment options, the etiology and pathophysiology of the disease remain poorly understood. Common risk factors include anatomical features including ulnar variance, differences in blood supply, increased intraosseous pressure along with direct trauma, and environmental influence. The staging of Kienböck’s disease depends mainly on radiographic characteristics assessed according to the modified Lichtman scale. The selection of treatment options is often challenging, as radiographic features may not correspond directly to initial clinical symptoms and differ among age groups. At the earliest stages of Kienböck disease, the nonoperative, unloading management is generally preferred. Patients with negative ulnar variance are usually treated with radial shortening osteotomy. For patients with positive or neutral ulnar variance, a capitate shortening osteotomy is a recommended option. One of the most recent surgical techniques used in Stage III Kienböck cases is vascularized bone grafting. One of the most promising procedures is a vascularized, pedicled, scaphoid graft combined with partial radioscaphoid arthrodesis. This technique provides excellent pain management and prevents carpal collapse. In stage IV, salvage procedures including total wrist fusion or total wrist arthroplasty are often required.

## 1. Introduction

Kienböck’s disease is described as avascular osteonecrosis of the lunate. The etiology of this disease remains unknown and controversial [1]. However, it is possible to determine factors that generally impact the risk of incidence. The anatomical risk factors include the shape of the lunate and distal radius, ulnar variance, the coverage of lunate by radius, the blood supply, the excessive intraosseous pressure, and the venous stasis [2]. Personal factors include age and gender, the associated diseases, the trauma-related factors, the social and environmental factors, and the association with osteonecrosis of other carpal bones [2]. Kienböck’s disease is classified as rare and its prevalence is about 7 per 100,000 [3,4]. Kienböck’s disease shows male predominance, with a peak incidence in patients aged 20–40 years [5]. Repetitive manual labor was reported as a risk factor but currently is recognized as a factor aggravating symptoms of an already established disease [6]. Usually one hand is affected; only 4% of cases of the disease are bilateral. It is known that there is an association between Kienböck’s disease and type 1 diabetes mellitus, systemic lupus erythematosus, and Legg-Calve-Perthes disease [5]. On examination, painful swelling on the dorsal side of the lunate can be observed. Decreased range of motion and grip strength of the wrist are typical [7]. Treatment of Kienböck’s disease varies, starting from conservative modalities to surgical procedures, although none of them is a gold standard. The algorithm of treatment depends on clinical and radiological symptoms [7]. The Lichtman scale is the most common radiological classification used to describe Kienböck’s disease. In 2003, Goldfarb and colleagues proposed a new, improved Lichtman scale [8]. The arthroscopic classification was developed by Bain et al. It is based on the number of articular surfaces of the lunate and adjacent articulation, which are defined as non-functional [9].

## 2. Staging

**Figure 1:**
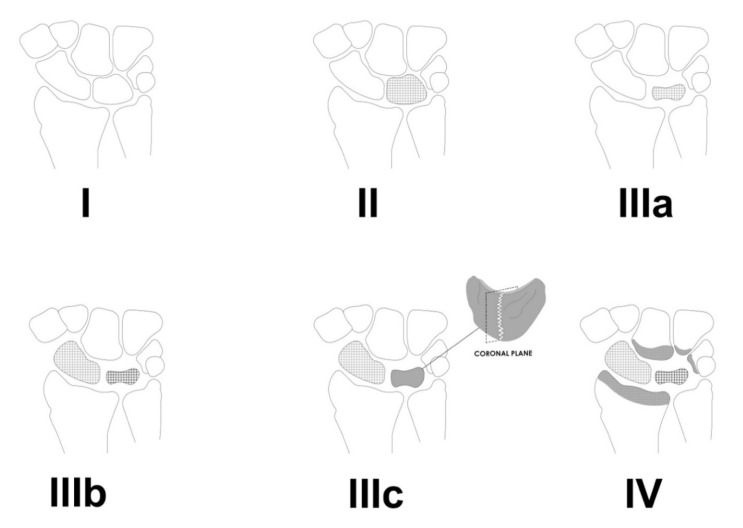
A graphic representation of different stages of Kienböck’s disease. In stage I lunate maintains normal architecture and density. Stage II is characterized by an increase in lunate density and diffused sclerosis of the lunate (visualized with a checkered pattern). In stage IIIa the lunate is collapsed but its carpal alignment and height remain unchanged (notice the decreased size of the lunate). In stage IIIb apart from lunate collapse, scaphoid palmar flexion occurs (visualized with a checkered pattern). Stage IIIc is reserved for complete coronal plane split (depicted as a bisection of the lunate). Stage IV is a combination of lunate collapse and radiocarpal or midcarpal degenerative arthritis (marked as grey areas on the articular surfaces of affected joints).

**Figure 2:**
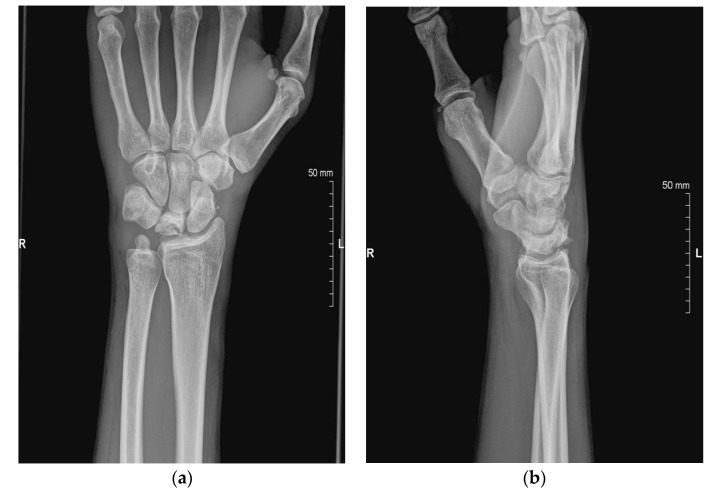
X-ray stage 3A: the collapse of lunate, carpal high preserved. (**a**) Stage 3A, AP view X-ray; (**b**) Stage 3A, lateral view X-ray.

**Figure 3:**
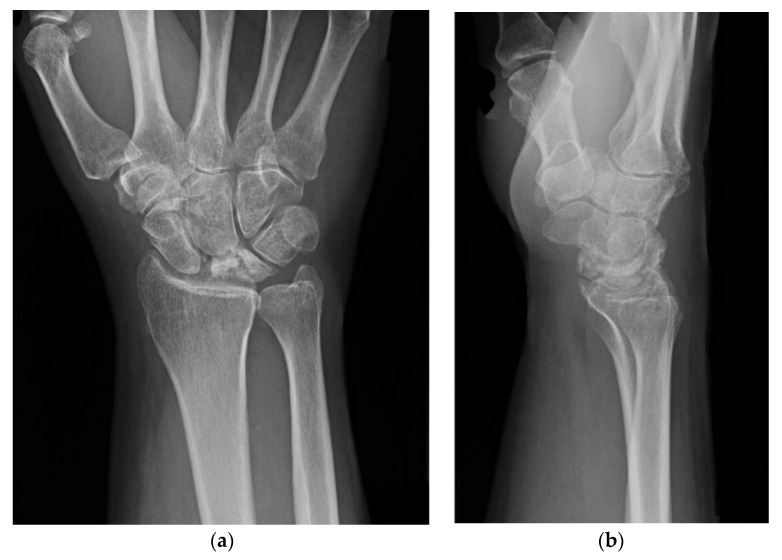
X-ray stage 3B: the collapse of lunate, carpal instability, scaphoid rotation, radioscaphoid angle (RSA) increased. (**a**) Stage 3B, AP view X-ray; (**b**) Stage 3B, lateral view -ray.

The staging of Kienböck’s disease depends mainly on radiographic evaluation [8]. However, vascularization (Schmitt) and cartilage (Bain) evaluation are also clinically relevant [10,11]. Proper classification of the disease is essential in choosing the right treatment for a patient with osteonecrosis of the lunate (Table 1, Figure 1). In stage I, the lunate maintains normal architecture and density, yielding normal plain radiographs [7]. However, on T1-weighted MR images, the signal is slightly decreased [5,12]. At this stage the lunate is often intensively enhanced after contrast application, reflecting bone marrow edema [5]. In Stage II, radiographs show increased lunate density and diffused sclerosis, however, the wrist’s architecture is not compromised [5,8]. In Stage III the lunate collapses. In this stage hyperintensity of the lunate marrow can be seen on fluid-sensitive T2-weighted images [5]. In 1993, stage III was divided into two stages: 3A and 3B, and it was further modified in 2010 with the addition of stage 3C [5]. In stage IIIA the lunate is collapsed but its carpal alignment and height remain unchanged [7]. Stage IIIB is associated with articular collapse, proximal migration of the capitate, and scaphoid palmar flexion, reflected by a cortical “ring sign” on radiographs [12] (Figure 2 and Figure 3). Stage IIIC is reserved for a complete coronal plane split, regardless of the lunate/wrist morphology [13]. Stage IV is a combination of lunate collapse and radiocarpal or midcarpal degenerative arthritis [13].

## 3. Treatment in Early Stages (Lichtman Stages I, II, and III-A)

The choice of treatment option for Kienböck’s disease can be difficult, primarily due to the variety of factors that ought to be considered in the treatment procedure [7]. These factors include the patient’s age, osseous assessment (Lichtman), ulnar variance, vascular assessment (Schmitt), and cartilage assessment (Bain) [14].

### 3.1. Stage I (Lichtman Stage I)

At the earliest stages of Kienböck disease, the management is usually nonoperative [14]. Immobilization with a splint or short arm cast (for at least three months) is the initial treatment option [8,14]. Even though Kienböck disease is a progressive disease, many patients treated conservatively stay functional with acceptable symptoms. Therefore, unless the patient remains symptomatic, no surgical interventions should be conducted [8]. In line with this notion, Brent et al. demonstrated a retrospective study involving 25 patients treated nonoperatively, where 12 wrists were in stage I. The study had a mean length of radiographic follow-up of 5.2 years and the observed progression in the Lichtman stage was 0.48 ± 0.09 stages per year. Importantly, despite the radiographic disease progression, nonoperational management improved pain, grip strength, and Mayo wrist score [15].

### 3.2. Stage II (Lichtman Stage II, Bain 0, Schmitt Stage A)

The three main treatment goals for stage II are lunate unloading, lunate decompression, and lunate revascularization [8,13,14]. Each patient with stage II should be matched with an individualized treatment method. Ulnar variance is a crucial factor that categorizes patients into two groups: negative-ulnar variance and neutral or positive ulnar variance [7]. Patients with negative ulnar variance are usually treated with radial shortening osteotomy [14]. The effectiveness of this method has been proven by Raven et al. in a study that involved 12 patients treated with radial shortening osteotomy with 22 years of follow-up. In this study a vast majority of patients had excellent pain relief and function [16]. A similar study with similar results has been conducted by Watanabe et al. with 13 patients and a follow-up of 21 years. In this study, 12 out of 13 patients noticed an improvement in terms of pain management [17]. However, according to Quenzer et al., radial shortening osteotomy combined with vascularized bone grafting achieves better results than joint-leveling procedure alone [18]. In line with this study, Daecke et al., with a long- term follow-up study, proved that radial shortening osteotomy along with vascularized pisiform bone grafting is an effective method in treating patients with negative ulnar variance [19]. Alternatively, the ulnar lengthening osteotomy can be considered. A few studies featuring long-term follow-up reported a good clinical outcome in patients treated with ulnar lengthening osteotomy [20,21]. In a biomechanical study conducted by Trumble et al., radial shortening osteotomy and ulnar lengthening achieved similar results regarding lunate strain [22]. Contrarily, the most recent study performed on cadavers showed more beneficial pressure distribution in preparations that underwent shortening of the radius compared to the lengthening of the ulna [23]. Overall, radial shortening osteotomy remains the most popular option regarding joint-leveling procedures [24]. This can be explained by the stronger scientific evidence favoring radial shortening and the occurrence of union-related complications associated with ulna lengthening [14,16,17,25]. For patients with positive/neutral ulnar variance, a capitate shortening osteotomy and radius wedge osteotomy are recommended options. Both of these methods mitigate revascularization by lunate unloading and achieving comparable clinical outcomes [13,14,26,27]. A different lunate-protective treatment method for stage II is lunate forage, which achieves lunate decompression [14]. It involves arthroscopic lunate drilling aimed at decreasing venous hypertension [28]. The advantage of this method is low invasiveness and therefore quick patient recovery. In a study involving 20 patients, treatment with lunate core decompression improved range of motion scores in all stage I and stage II cases [29]. Recently, a prospective cohort study involving 82 patients proved that arthroscopic lunate core decompression achieves comparable results to radial shortening osteotomy [30].

Apart from lunate unloading and lunate decompression, revascularization procedures play a vital role in treating stage II. The revascularization can be direct or indirect. The most notable indirect method of revascularization is core decompression of the distal radius/ulna [31]. The surgeon curettes cancellous bone of the distal metaphysis in the radius and ulna or in just the radius. This procedure initiates sufficient hyperemia for lunate revascularization. A series of studies conducted by Illarramendi et al. involving patients that underwent metaphyseal core decompression of both ulna and radius presented satisfying long-term follow-up results [32,33]. In line with this, 10 out of 12 patients in stages I and II that were treated with just radial metaphyseal core decompression achieved satisfactory midterm outcomes [34]. Overall, metaphyseal core decompression is an extra-articular and relatively simple operation with no reported complications [34]. Direct vascularization of the lunate can be achieved with a pedicled graft or free vascularized bone graft (VBG) [11]. VBG is the most frequently used method of treating stage II with neutral ulnar variance and the third most frequently used in stage I (in patients who failed immobilization) in the United States [24]. The popularity of this procedure is due to the amount of research that has proven the effectiveness of revascularization techniques [1,19,35,36,37]. The most popular VBG technique among American hand surgeons is VBG using the fourth and fifth extensor compartment arteries (ECA) [24]. The treatment aims at directing the fifth ECA with the retrograde flow into the 4th ECA with orthograde flow providing revascularization and remodeling of the avascular lunate [28]. Apart from the above-mentioned procedure, a vascularized pisiform bone transfer is also effective. Daecke et al., in their study of 23 patients with a mean follow-up of 12 years, revealed excellent results from this procedure. In 20 out of 23 patients there was a significant decrease in pain [19]. Other bone graft methods include volar radial graft, medial femoral condyle graft, the 1–2 inter compartmental artery, and the second dorsal metacarpal neck grafts [37,38]. In the most recent systematic review, which included 92 VBG-treated patients with Kienböck disease, all subjects achieved improvement in grip strength and pain with no effect on the range of motion. In cases treated with 4 + 5 ECA graft, 71% achieved lunate revascularization, and in 15%, progression of the disease was observed within a mean follow-up of 45 months [38].

It is important to mention that direct revascularization procedures are relatively more challenging for the surgeon and can result in the damaging of the articular surfaces due to the intra-articular nature of such treatment. Furthermore, lunate unloading/decompression-oriented procedures are simpler and offer comparable results.

### 3.3. Stage IIIA (Lichtman Stage IIIA, Bain 1, Schmitt Stage B)

At this stage the lunate is compromised, so the goal of the treatment should be set on lunate reconstruction via revascularization and/or unloading [14,25]. Of note, Patients in IIIA with negative ulnar variance benefit from joint leveling procedures similarly to patients in stage II [16]. Recently, an interesting approach was presented by Hong et al. in a study, where 18 patients in stage IIIA underwent radial wedge osteotomy in combination with radius shortening osteotomy. In this study, 16 cases of stage IIIA and two cases of stage IIIB achieved improvement regarding the range of motion of the wrist and pain. This method theoretically would allow patients with non-negative ulnar variance to benefit from radius shortening osteotomy similarly to patients with negative ulnar variance. Despite the promising results, a significant limitation of this study is the short follow-up period (22.3 months), thus lacking data covering disease progression rate/late occurring complications [39]. The recommended technique by Lichtman et al. is a vascularized medial femoral trochlea (MFT) graft [14]. Higgins et al. demonstrated satisfactory results in performing the MFT graft in patients at stage IIIA. In this study, in 15 out of 16 patients, CT affirmed healing [18]. Illarmendi et al. presented results from the treatment of 25 patients in stage IIIA with radius core decompression. Although satisfying results could be observed in most of the patients, the results were not as good as in patients in earlier stages [33]. However, more recently de Carli et al. presented a study that included 15 patients in stage IIIA that received radius core decompression. Fourteen out of 15 patients returned to their original employment and only two wrists progressed to later stages of the disease [17]. Apart from VBG, other viable surgical techniques are available such as proximal row carpectomy radioscapholunate fusion or scaphocapitate fusion [6,7,8]. The alternatives do not provide lunate reconstruction, and therefore are more destructive than lunate reconstruction-oriented procedures.

## 4. Surgical Procedures in Late-Stage Patients (Lichtman IIIB, IIIC, and IV)

There is a wide range of procedures that have been developed for the treatment of late-stage Kienböck disease throughout the years (Table 2). However, retrospective systemic reviews proved that none of the treatment options was superior concerning pain, grip strength, or motion measures [7]. Therefore, the most recent treatment algorithm recommends a highly individualized approach, taking the patient’s age, state of the lunate, and state of the wrist into consideration [14].

In age groups younger than 15 years of age, those 16–20, and older than 70, non-operative treatment is advisable in the first place regardless of the stage of the disease [14].

### 4.1. Stage IIIB, IIIC

This stage is characterized by the degeneration of the lunate articular surface and loss of the carpal height due to scaphoid flexion (radioscaphoid angle > 60) [53]. Because of the maintenance of radioscaphoid articulation, one of the main surgical procedures performed is Scaphocapitate (SC) fusion. The main target of this procedure is to stabilize the midcarpal joint and unload the lunate, preventing it from collapsing [8].

Alternatively to SC fusion, scaphotrapezio-trapezoid (STT) fusion can be performed [14]. Unfortunately, the effectiveness and reliability of these methods are differentiated. Early studies demonstrated satisfactory clinical results of performing STT procedures in Lichtman stage III patients [42]. However, recent articles showed that the STT procedure may cause severe pain, partial loss of mobility, and longer rehabilitation time compared to non-invasive treatment [54,55]. Radioscapholunate (RSL) arthrodesis is a partial fusion procedure which preserves the motion of the midcarpal joint and can provide significant pain relief. This procedure is particularly recommended in patients with arthritis of the metacarpal joint [43].

Proximal Row Carpectomy (PRC) is an old but still effective salvage procedure provided that the capitate head is well-preserved and the lunate fossa of the radius is in perfect condition [56]. Dorsal capsule interposition can also be performed in patients with mild arthritic changes of the capitate [51]. However, a recent randomized trial suggests that a dorsal capsule flap does not improve the range of motion and functional range in PRC [57]. Numerous studies with long-term follow-up of a minimum of 10 years have demonstrated that PRC is a reliable long-term treatment approach for stage III and IV Kienböck disease [44,45,46]. Nonetheless, the use of PRC in stage IV cases should be limited due to the risk of early degeneration of capitate and radius articular surfaces. PRC with distal radius hemiarthroplasty is a novel procedure dedicated to patients with wrist arthritis [58].

One of the most recent surgical techniques used in Stage III Kienböck cases is Vascularized Bone Grafting (VBG). Until now, the pedicled osseous grafts have been transposed from the ulna, ulnar shaft, pisiform, radial metaphysis, and scaphoid [59,60,61,62,63,64]. Moreover, free vascularized bone transfers from the medial femoral condyle and iliac crest have been described [1,65,66]. The most significant component of these procedures includes the preparation of tension-free vascular pedicles, which include nutrient vessels supplying both cancellous and cortical parts of the bone [67]. The majority of follow-up or postoperative studies demonstrated excellent reduction of pain, increased wrist mobility, and improvement of strength [68]. In particular, the long-term effects of free vascularized iliac bone transfer in a late-stage Kienböck’s disease study found a major improvement in the flexion-extension arc, grip strength, pain, DASH score, Green O’Brien score, and Stahl index [1]. Bone marrow transfusion from distal radius or iliac crest combined with low-intensity pulsed ultrasound may be a less invasive alternative to VBG, with promising results regarding pain and functional score six years after the procedure [49,69].

**Figure 4:**
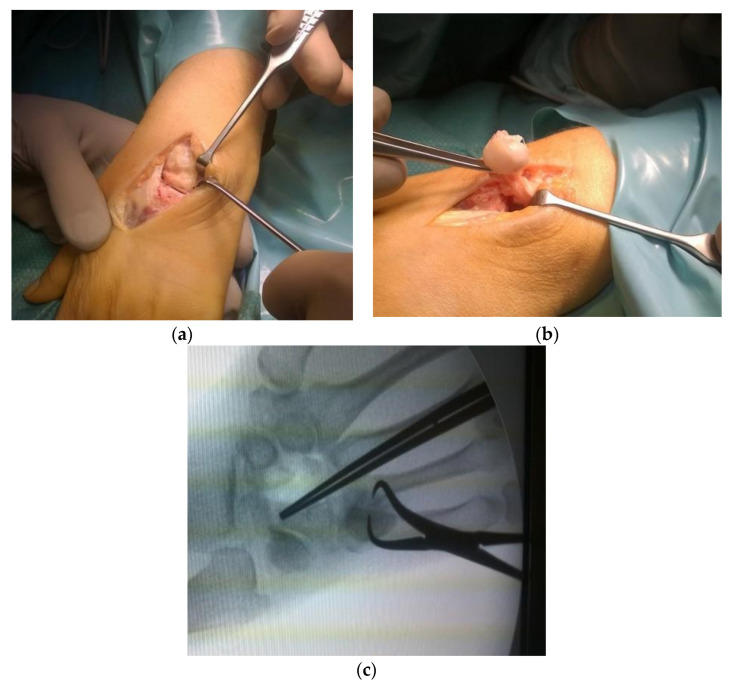
Photographs were obtained during surgery in a patient undergoing vascularized capitate transposition and capitate osteotomy combined with the vascularized bone transfer (modified Granner’s method). (**a**) A view of the anatomy of the radiolunate joint fully exhibited using a dorsal surgical approach; (**b**) Vascularised capitate graft being harvested; (**c**) X-ray of the wrist after lunate excision and transposition of the capitate.

Another perspective method in late-stage Kienböck’s disease is vascularized capitate transposition +/− capitate osteotomy combined with the vascularized bone transfer (Figure 4). Short-term follow-up studies demonstrated promising clinical results, but long-term follow-up results are still required [47,48].

Lunate excision performed alone or preferably combined with STT provided pain relief and improvement in wrist mobility. Nevertheless, the tendency of scaphoid shifting towards lunate fossa was observed [70]. Lunate replacement techniques were also developed, using silicone, titanium, pyrocarbon, or 3D printed prostheses with a view to stabilizing the wrist and preserving its mobility [67,71,72]. 3D printed, personalized prostheses show favorable results in short to mid-term follow-ups of preliminary studies [73]. However, the use of these methods is limited owing to the lack of long-term follow-up studies and the inherent instability of prostheses [67]. Lunate excision with replacement could be particularly beneficial in Lichtman stage IIIC cases when coronal plane split and partial lunate collapse occur without the possibility of revascularization or reconstruction. However, modification to lunate replacement methods is required to obtain satisfactory clinical outcomes [14]. Arthroscopic excision of the lunate without replacement may be a viable alternative for low-demand patients in need of clinical and functional improvement in the short to mid-term [74]. Inevitable complications in a longer follow-up period limit the use of this method across other patient populations.

### 4.2. Stage IV

In this stage, due to total lunate collapse with nonfunctional radioscaphoid articulation and advanced arthritic changes in the capitolunate joint, the wrist is often no longer reconstructable. Therefore, salvage procedures in stage IV are preferred. These procedures include limited or total wrist arthrodesis and total wrist arthroplasty [14,50]. Alternatively, PRC with dorsal wrist capsule arthroplasty can be performed. Nonetheless, advanced arthritic changes may lead to failure of this procedure and reduced grip strength [51]. Additional wrist denervation can be beneficial in symptom alleviation. A long-term study of patients with stage IV Kienböck disease, with an average of 9.6 years follow-up, proved that complete wrist denervation resulted in subjective improvement in two-thirds of patients. Approximately one-half of the patients admitted complete or considerable alleviation of pain [52]. There are also a limited number of described late-stage disease cases (including one patient with stage IV) treated with total lunectomy and lunate replacement with a 3D printed, personalized, titanium–alloy lunate prosthesis [73].

## 5. Adjuvant Procedures

A handful of additional procedures can be performed autonomously or in combination with other described procedures to improve the outcome of treatment [67]. Arthroscopic or open synovectomy is especially beneficial, considering that synovitis is one of the typical findings in Kienböck’s disease [8,9]. Avik et al. also proposed arthroscopic debridement and arthrolysis as alternative treatment options for salvage procedures in late–stage disease. These treatment approaches resulted in excellent pain management but failed to show a significant increase in wrist motion and grip strength [75]. Furthermore, temporary STT joint fixation can unload the lunate, thus promoting revascularization. This procedure is applied primarily in conjunction with VBG and as the first-choice procedure in teenage patients with Kienböck’s disease [53,76]. Recently, the use of cultured autologous multipotent mesenchymal stromal cells in Kienböck’s disease was reported. However, this method is in the experimental stage and its long-term effectiveness is unknown [77].

## 6. Summary

The ideal treatment for different stages of Kienböck’s is still under debate. Furthermore, clear guidelines based on randomized multicenter, controlled studies are missing. Choosing the right treatment method is clinically demanding for hand surgeons. The algorithm should depend primarily on the clinical symptoms, radiological evaluation, and the surgeon’s experience. The Lichtman scale is the most common radiological classification used to describe Kienböck’s disease. Proper classification of the disease is essential and is the main determinant in choosing the right treatment for a patient with osteonecrosis of the lunate. However, the choice of procedure should also take into account anatomical and personal factors. Based on retrospective data, no management of Kienböck’s disease has sufficient data to determine that the intervention outcomes are the most effective. This review explored the advantages and disadvantages of the available up-to-date treatment options and their matching to the best indications. At the earliest stages of Kienböck disease, nonoperative management is predominantly advised. The main goal in treating stage II is revascularization, unloading, and decompression of the lunate. Stage IIIA often requires lunate reconstruction, while IIIB and IIIC frequently involve partial wrist arthrodesis. Salvage procedures such as wrist arthrodesis are limited mainly to Lichtman IV.

## Figures and Tables

**Table 1:** Lichtman’s classification.

Stage	Radiographs	MRI
I	Normal	↓ T1 signal, lunate enhancement after contrast administration
II	Increased density without lunate collapse	↓ T1 signal, variable T2 signal
IIIA	Lunate collapse, Radioscaphoid angle < 60°	↓ T1 signal, variable T2 signal
IIIB	Lunate collapse with scaphoid palmar flexion (radioscaphoid angle > 60°)	↓ T1 signal, variable T2 signal
IIIC	Lunate collapse with coronal lunate fracture (chronic)	↓ T1 signal, variable T2 signal
IV	Lunate collapse with radiocarpal or midcarpal degenerative arthritis	↓ T1 signal, variable T2 signal

The downward arrow represents decrease in T1 signal.

**Table 2:** Recommended treatment strategies for each stage of Kienböck disease.

Stage of Kienböck’s Disease in Lichtman Scale	Sub-Stage	Leading Treatment	
		Aim of Treatment	Procedure
I		Preventing progression [14]	Usually nonoperative (immobilization with a splint or short arm cast for at least three months) [14,15]
II	With a negative ulnar variance	Lunate unloading, decompression and revascularization [14]	radial shortening osteotomy (selectively or with vascularized pisiform bone grafting) [16,17,18] or lunate core decompression [29,30]
	With a positive or neutral ulnar variance	Lunate unloading, decompression and revascularization [14]	capitate shortening osteotomy or radial closing wedge osteotomy [26,27] or lunate core decompression [29,30] And/or revascularization indirect: radial core decompression [33,34] or direct: VBG 4 + 5 ECA [37,38]
III	A	Lunate reconstruction through lunate unloading and revascularization [14,25]	With negative ulnar variance: radial shortening osteotomy [16,17,18] and/or revascularization indirect: radial core decompression [33,40] or direct: VBG 4 + 5 ECA [37,38] or Vascularized medial femoral trochlea graft (MFT) [41] With positive or neutral ulnar variance: radial closing wedge osteotomy [26] and/or revascularization indirect: radial core decompression [33,40] or direct: VBG 4 + 5 ECA [37,38]
	B	Preventing carpal collapse [8]	Choice according to a highly individualized approach among: - Scaphocapitate (SC) fusion [8] - Scaphotrapezio-trapezoid (STT) Fusion [42] -Radioscapholunate (RSL) arthrodesis [43] - Proximal Row Carpectomy (PRC) [44,45,46] - Vascularized capitate transposition +/− capitate osteotomy combined with the vascularized bone transfer (modified Granner’s method) in late-stage Kienböck’s disease [47,48] - Bone marrow transfusion combined with low-intensity pulsed ultrasound [49]
	C	Preventing carpal collapse [8]	
IV		Salvage procedures [14,50]	- Total or limited wrist arthrodesis or total wrist arthroplasty [14,50] - PRC with dorsal wrist capsule arthroplasty [51] + Additional wrist denervation can be beneficial in symptom alleviation [52]
In age groups <20 y/o and >70 y/o non-operative treatment is advisable in the first place regardless of the stage of the disease [14]			

## Data Availability

Not applicable.
